# Detection and Molecular Characterization of Porcine Parvovirus 7 in Eastern Inner Mongolia Autonomous Region, China

**DOI:** 10.3389/fvets.2022.930123

**Published:** 2022-07-06

**Authors:** Shubo Wen, Yang Song, Xiangyu Lv, Xiaogang Meng, Kai Liu, Jingfeng Yang, Fengying Diao, Jinfei He, Xiaowei Huo, Zeliang Chen, Jingbo Zhai

**Affiliations:** ^1^Preventive Veterinary Laboratory, College of Animal Science and Technology, Inner Mongolia Minzu University, Tongliao, China; ^2^Brucellosis Prevention and Treatment Technology Research Center, Inner Mongolia Autonomous Region, Tongliao, China; ^3^Key Laboratory of Zoonose Prevention and Control at Universities of Inner Mongolia Autonomous Region, Tongliao, China

**Keywords:** porcine parvovirus 7, phylogenetic analysis, molecular epidemiology, recombination, mutation

## Abstract

Porcine parvoviruses (PPV) and porcine circoviruses type 2 (PCV2) are widespread in the pig population. Recently, it was suggested that PPV7 may stimulate PCV2 and PCV3 replication. The present study aimed to make detection and molecular characterization of PPV7 for the first time in eastern Inner Mongolia Autonomous Region, China. Twenty-seven of ninety-four samples (28.72%) and five in eight pig farms were PPV7 positive. Further detection showed that the co-infection rate of PPV7 and PCV2 was 20.21% (19/94), and 9.59% (9/94) for PPV7 and PCV3. In addition, the positive rate of PPV7 in PCV2 positive samples was higher than that in PCV2 negative samples, supporting that PCV2 could act as a co-factor for PPV7 infection. In total, four PPV7 strains were sequenced and designated as NM-14, NM-19, NM-4, and NM-40. The amplified genome sequence of NM-14 and NM-40 were 3,999nt in length, while NM-19 and NM-4 were 3,996nt with a three nucleotides deletion at 3,097–3,099, resulting in an amino acid deletion in the Cap protein. Phylogenetic analysis based on the capsid amino acid (aa) sequences showed that 52 PPV7 strains were divided into two clades, and the four PPV7 strains in this study were all clustered in clade 1. The genome and capsid amino acid sequence of the four PPV7 strains identified in this study shared 80.0–96.9% and 85.9–100% similarity with that of 48 PPV7 reference strains selected in NCBI. Simplot analysis revealed that NM-19 and NM-4 strains were probably produced by recombination of two PPV7 strains from China. The amino acid sequence alignment analysis of capsid revealed that the four PPV7 strains detected in Inner Mongolia had multiple amino acid mutations in the 6 B cell linear epitopes compared with the reference strains, suggesting that the four PPV7 strains may have different characteristics in receptor binding and immunogenicity. In summary, this paper reported the PPV7 infection and molecular characterization in the eastern of Inner Mongolia Autonomous Region for the first time, which is helpful to understand the molecular epidemic characteristics of PPV7.

## Introduction

The parvoviruses, classified taxonomically within the family *Parvoviridae*, are small, non-enveloped, single-strand linear DNA viruses, that are capable of infecting many animals (1). *Parvoviridae* is divided into two subfamilies: *Parvovirinae* and *Densovirinae*, which infect vertebrates and arthropods. Currently, the *porcine parvovirus* contains nine genera, of which *Protoparvovirus, Teraparovirus, Bocaparvovirus, Copiparvovirus*, and *Chapparvovirus* infect pigs (2, 3). The genome of parvovirus is 4–6.3 kb in length. PPV1, belonging to *Protoparvovirus*, is considered one of the main pathogens associated with abortion worldwide. It was first isolated in 1965 from a cell culture contaminant in Germany (4, 5). In the following years, PPV was identified to cause recurring oestrus, abortion, and the delivery of mummified or stillborn fetuses, commonly described as SMEDI (stillbirth, mummification, embryonic death, and infertility). The virus is believed to be endemic in most regions of the world and can be detected in all pig herd categories.

With the development of next-generation sequencing techniques, six novel PPV genotypes were identified subsequently (3, 6–8). These *Porcine Parvovirus* species are taxonomically divided into four genera: *Protoparvovirus* (PPV1), *Tetraparvovirus* (PPV2-3), *Copiparvovirus* (PPV4-6), and *Chapparvovirus* (PPV7) based on the amino acid similarity of the NS1 protein (2). Among them, the most recently reported PPV7 was initially identified from healthy pigs' rectal swab samples using metagenomic sequencing in America in 2016. Subsequently, PPV7 was detected in Sweden (9), Poland (10), Korea (11), China (12), Brazil (13), and Colombian (14).

The genome of PPV7 is ~4,000 bp in length and contains two major open reading frames (ORFs), the ORF1 and the ORF2. The ORF1 encodes non-structural protein 1 (NS1) responsible for viral replication. While the ORF2 encodes a structural protein (Cap) (3), the major antigenic component in PPVs, capable of inducing neutralizing antibodies against viral infection (15). The pathogenicity of PPV7 has not been determined yet, however, the presence of PPV7 in aborted fetuses and semen indicates that PPV7 may be associated with reproductive failure.

In China, PPV7 was first reported in Guangzhou, and then in Anhui, Guangxi, Hunan, Jiangsu, Hebei, and Shandong provinces (12, 16–20). However, there is limited genetic information on PPV7 in Northern China. In this study, we aim to detect and make molecular characterization of PPV7 in eastern Inner Mongolia Autonomous Region, China for the first time.

## Materials and Methods

### Sample Collection and DNA Extraction

**Figure 1:**
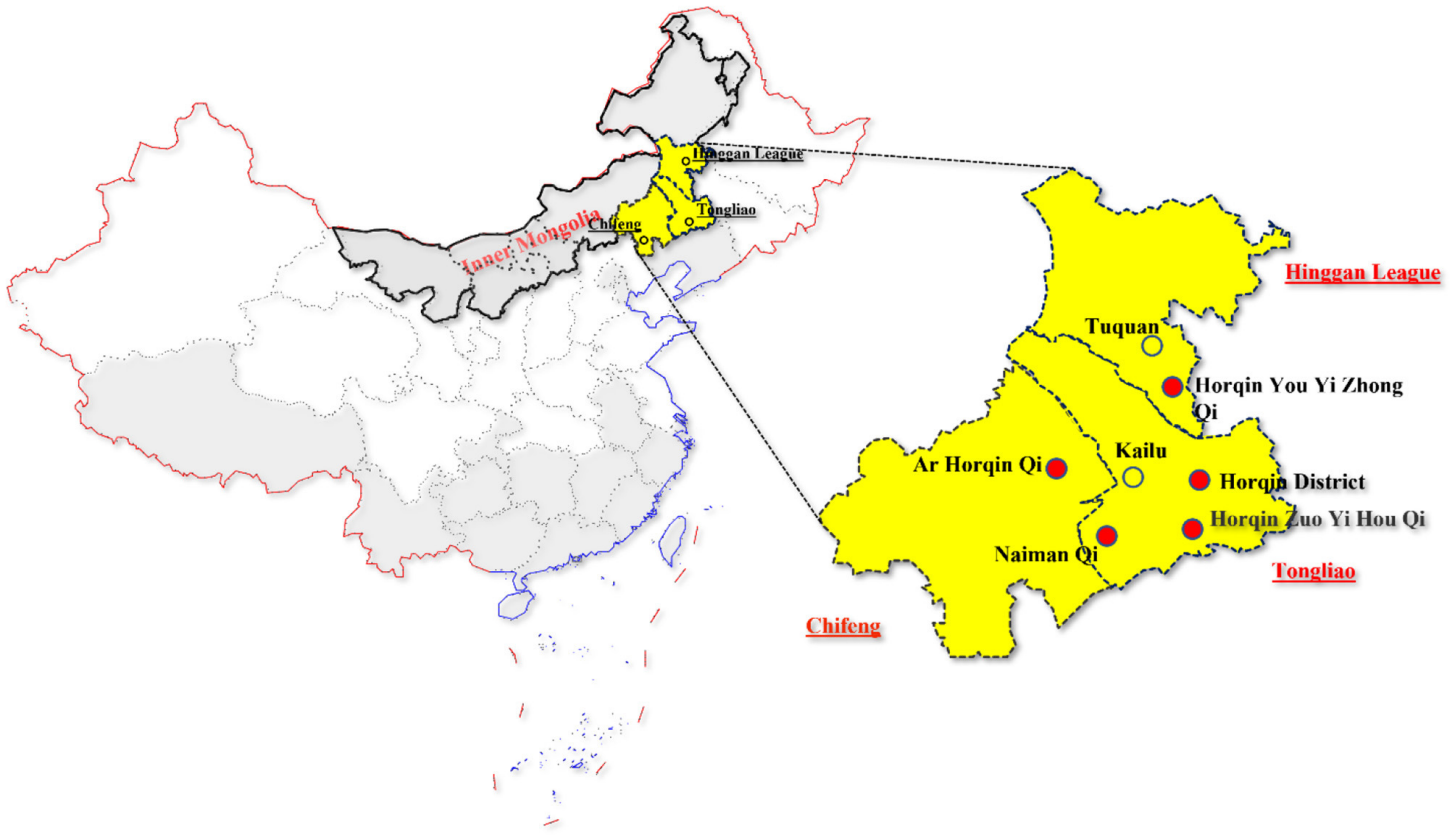
Geographical information for tissue samples collected in Inner Mongolia, China. The geographical locations of the samples were marked in yellow. The solid red circles are the locations of farms that tested positive for PPV7 and the hollow circles are the locations of farms that tested negative for PPV7.

Ninety-four lung samples of diseased pigs from 8 commercial pig farms in seven counties of Tongliao, Hinggan League, and Chifeng in eastern Inner Mongolia (Figure 1) were collected by our lab in 2020. The sick pigs suffered from different clinical symptoms, mainly gastrointestinal or respiratory disease. A total of ninety-four lung homogenates were prepared and stored in a freezer at −80°C for further DNA extraction. Total genomic DNA from tissue samples was extracted using the TIANamp Virus DNA Kit (TIANGEN BIOTECH, Beijing, China) as our previous publication (21).

### Detection of PPV7, PCV2, PCV3 and PCV4

All the collected samples were detected for PPV7, PCV2, PCV3, and PCV4 using primers listed in Table 1. The primers for PCV2, PCV3, and PCV4 detection were all synthesized according to previous studies (21, 22). The PPV7-2F/2R were designed for PPV7 detection and genome amplification. In this study, 25 μl PCR (Polymerase chain reaction) system was applied, containing 22 μl T3 Super PCR Mix (Tsingke Biotechnology, Beijing, China), 1 μl of 10 μM each of the two primers, and 1 μl genomic DNA. The following PCR reaction conditions were used: pre-denaturation at 98°C for 2 min, 35 cycles of denaturation at 98°C for 10 s, annealing at 58°C for 15 s, extension at 72°C for the 20 s, and a final extension at 72°C for 5 min. Finally, the PCR products were analyzed by 1.5% agarose gel electrophoresis.

**Table 1:** List of primers used in this study.

**Primer**	**Sequence (5'-3')**	**Amplicon length (bp)**
PPV7-1F	GGAACGACAAGGACGACACTT	1,282
PPV7-1R	CCCAGGCAGTTCTTGACGAT	
PPV7-2F	ACACAAGCCGGGATTCCAGCA	993
PPV7-2R	CCACGAGCACTCCATCCCCTC	
PPV7-3F	CGCAAGACTTGGCTTCAGCAC	1,045
PPV7-3R	GGATGCTGTCCGGGTTGGTGA	
PPV7-4F	CACCCGAGACGAACTGGAC	954
PPV7-4R	TGGCGTTGAGAAGACACTGGTTTAG	
PCV2-F	GGACCCCAACCACATAAAA	555
PCV2-R	CCCTAACCTATGACCCCTATGT	
PCV3-F	TCCAAACTTCTTTCGTGCCGTAG	264
PCV3-R	GGCTCCAAGACGACCCTTATGC	
PCV4-F	GTTTTTCCCTTCCCCCACATAG	391
PCV4-F	ACAGATGCCAATCAGATCTAGGTAC	

### PCR Amplification for PPV7 Full-Length Genomes

The genomic DNA was used as a template to amplify the viral genome of PPV7 by using high fidelity polymerase. Four primer pairs were designed and synthesized based on the reference sequences of isolate 42 (GenBank No. KU563733). The PCR mixture for viral genome amplification contained 1 μl TransStart FastPfu Fly DNA Polymerase (Transgene, Beijing, China), 1 μl of 10 μM each of the two primers, 5 μl Buffer, 2 μl 2.5 mM dNTPs, 14 μl dd H2O, and 1 μl genomic DNA. The PCR reaction was performed under the following conditions: pre-denaturation at 95°C for 2 min, 35 cycles of denaturation at 95°C for the 20 s, annealing at 56°C for 20 s, extension at 72°C for 30 s, and a final extension at 72°C for 5 min. The amplicons were separated using 1.5% agarose gel electrophoresis, purified with Gel Extraction Kit, and cloned into a pEASY-Blunt vector (Transgene, Beijing, China) according to the manufacturer's instructions. Four strains of PPV7 were successively cloned and named NM-14, NM-19, NM-4, and NM-40. Additionally, three recombinant amplifications of every strain were sequenced in both directions using the ABI 3730XL sequencer at Sangon (Nanjing, China). The genome sequences were assembled utilizing the software SeqMan (DNA-STAR Inc.). The nearly complete genome of the four PPV7 isolates were deposited in the GenBank under the accession numbers from OL856075–OL856078, respectively.

### Genome Analyses

Forty-eight nearly complete genomes of PPV7 strains at the GenBank were selected from different origins in different years and downloaded. The phylogenetic relationships were assessed by MEGA v7.0.14 with a p-distance-based, neighbor-joining method (bootstrap analysis with 1,000 replicates). Similarity analyses among the strains including the four PPV7 strains identified in this study were performed using the Lasergene Package (DNA-STAR Inc.) with the Clustal W method. A similarity plots analysis of 2 PPV7 isolates in this study, and 3 PPV7 strains GX22-1999 (MN326288), PPV7-HeN10-2015 (MN326255), and PPV7-XZ7-1999 (MN326252) was performed by the sliding window of 200 nucleotides (nt) and a step size of 20 nt. The other options, including Kimura (2-parameter) distance method, 2.0 Ts/Tv ratio, Neighbor-Joining tree model, 1,000 Bootstrap replicates were used. as implemented in the SimPlot, v. 3.5.1 package. The similarity plot of these PPV7 strains was drawn, and the PPV7 strains NM-19 and NM-4 were set as a query strain, respectively. Amino acid sequence alignment was made with the Lasergene Package (DNA-STAR Inc.). Antigenicity testing was done with the VaxiJen v2.0 server online tool (23) (VaxiJen v2.0, http://www.ddg-pharmfac.net/vaxijen/VaxiJen/VaxiJen.html).

### Statistical Analyses

All statistical analyses were performed by GraphPad Prism version 8.0.0 for Windows (San Diego, CA, USA) and SPSS 21.0 software (SPSS Inc., Chicago, IL, USA). The *t*-tests (parametric tests) were used for statistical comparison between the PPV7 positive rates in PCV2 positive samples and PCV2 negative samples, as well as in PCV3 positive samples and PCV3 negative samples. The *P*-value was adjusted with Benjamini-Hochberg approach for false discovery rate (FDR) with statistical significances declared at *P* < 0.05. All the data for statistical analyses were infection rates of PPV7, PCV2 and PCV3 in the five pig farms that detected positive for PPV7.

## Results

### Prevalence of PPV7 and Co-infections With PCV2, PCV3, and PCV4

**Figure 2:**
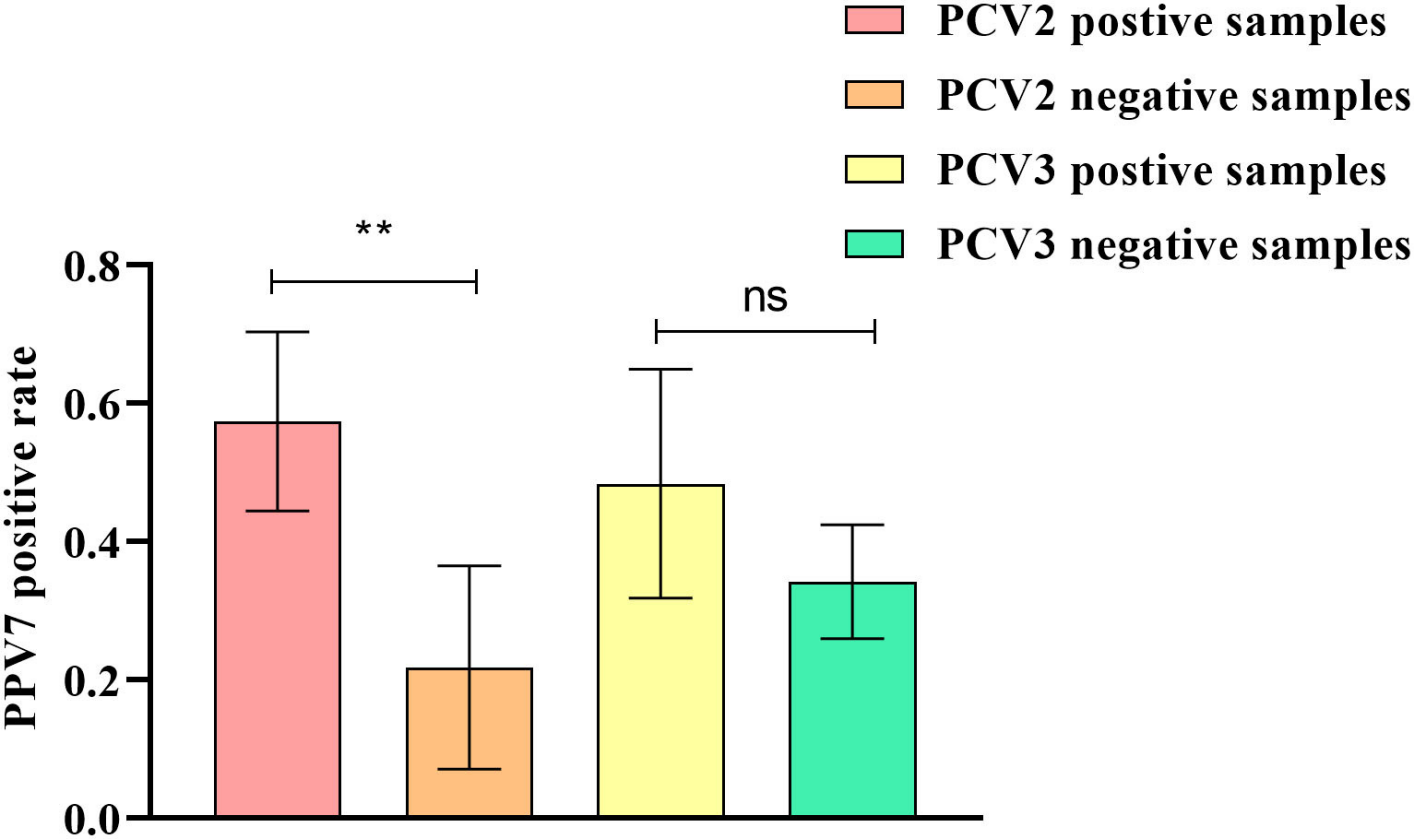
Co-infection of PPV7 with PCV2 and PCV3. The PPV7 infection rates in PCV2 positive samples were significantly higher than that in PCV2 negative samples. However, there is no significant difference between the PPV7 infection rates in PCV3 positive samples and PCV3 negative samples. **P < 0.01, ns *P* > 0.05.

In total, five in eight pig farms were detected positive for PPV7 in this study. The positive rates of PPV7, PCV2, and PCV3 at the individual sample level were 28.72% (27/94), 41.48% (39/94), and 24.47% (23/94), respectively. Meanwhile, none of the samples was detected positive for PCV4. The co-infection rate of PPV7 and PCV2, PPV7 and PCV3, as well as PPV7, PCV2, and PCV3 were 20.21% (19/94), 9.57% (9/94), and 9.57% (9/94). Interestingly, PPV7 can be detected in 48.71% (19/39) of PCV2 and 39.13% (9/23) of PCV3 positive samples, respectively. While, the PPV7 positive rates in PCV2 negative and PCV3 negative samples were 14.54% (8/55) and 25.35% (18/71), respectively. Of note, the PPV7 infection rates in PCV2 positive samples were significantly higher than that in PCV2 negative samples (*P* = 0.0037 <0.05). Whereas, there is no significance between the PPV7 infection rates in PCV3 positive samples and PCV3 negative samples (*P* = 0.125 > 0.05) (Figure 2). The details were listed in Table 2.

**Table 2:** Co-infection of PPV7 with PCV2 or with PCV3 in pig farms that are detected positive for PPV7.

**Farm**	**PPV7 positive rate**	**PPV7 positive rate in samples of**			
		**PCV2 positive**	**PCV2 negative**	**PCV3 positive**	**PCV3 negative**
A	7/18	6/12	1/6	2/5	5/13
B	6/12	4/7	2/5	3/5	3/7
C	3/13	3/6	0/7	1/4	2/9
D	7/16	4/5	3/11	2/3	5/13
E	4/12	2/4	2/8	1/2	3/10

### Complete Genome Sequencing and Phylogenetic Analysis of PPV7

To evaluate the molecular and evolutionary characteristics of PPV7 in eastern Inner Mongolia, China. Four PPV7 strains from diseased pigs were randomly selected and amplified for the nearly complete genome using the primers shown in Table 1. The occurrence of PPV7 in eastern Inner Mongolia of China was confirmed through genomic sequencing. The genomes of NM-14, NM-19, NM-4, and NM-40 were 3,999, 3,996, 3,996, and 3,999 nt in length, which contain two major ORFs encoding NS1 and Cap proteins, respectively. Compared with the genome of NM-14 and NM-40, a three-bases deletion occurred in the genome of NM-19 and NM-4 at positions 3,097–3,099, leading to an amino acid deletion in the Cap protein.

**Figure 3:**
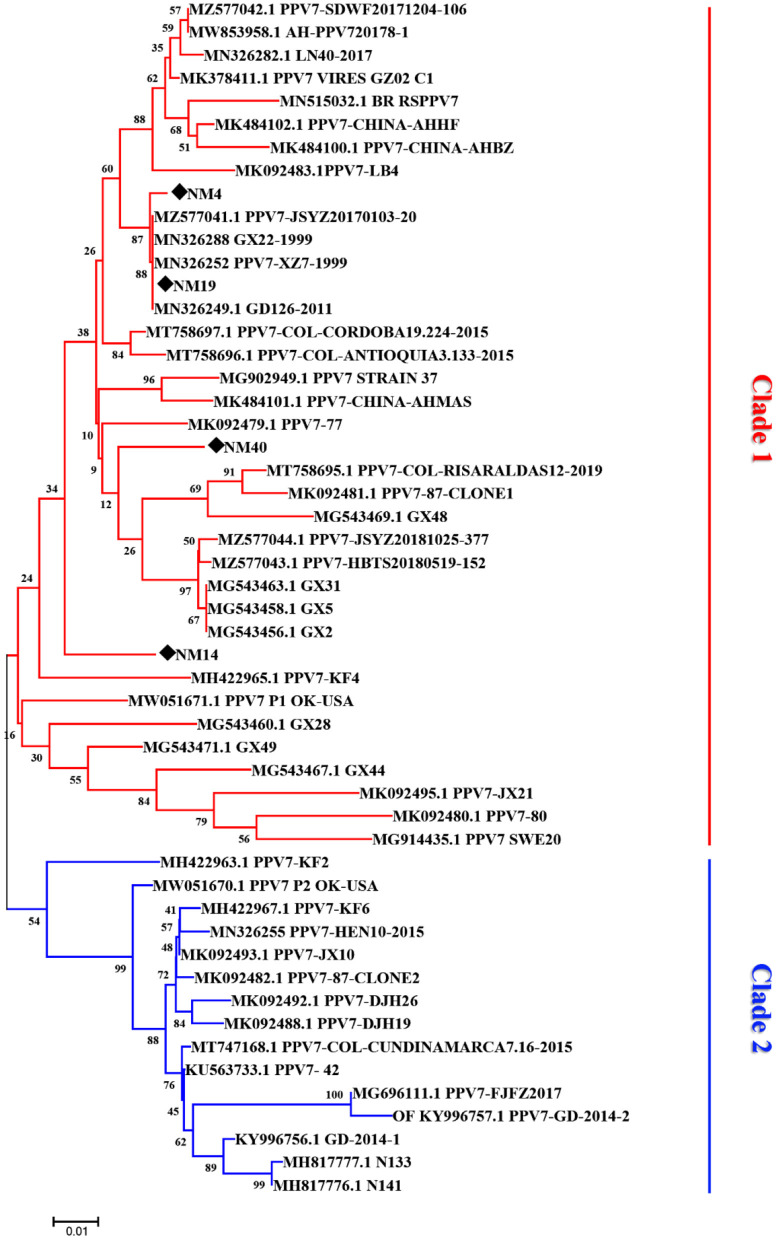
Phylogenetic tree based on the amino acid sequences of different porcine parvovirus 7 strains. The tree reliability was assessed using the Neighbor-joining method with the Poisson model and 1,000 bootstrap replications. The strains detected in our laboratory are marked with a black solid diamond.

The phylogenetic analysis based on the amino acids sequence of capsid showed that the 48 PPV7 strains selected in the GenBank and four PPV7 strains identified in this study were well-differentiated into two distinct clades (Figure 3). The four PPV7 strains in this study were all clustered into clade 1. In addition, NM-4 and NM-19 were closely related to JSYZ20170103-20, GX22-1999, PPV7-XZ7-1999, and GD126-2011. While NM-40 and NM14 were far related to other reference strains and were unique in eastern Inner Mongolia.

### Similarity Analysis and Recombination Analysis of PPV7

**Figure 4:**
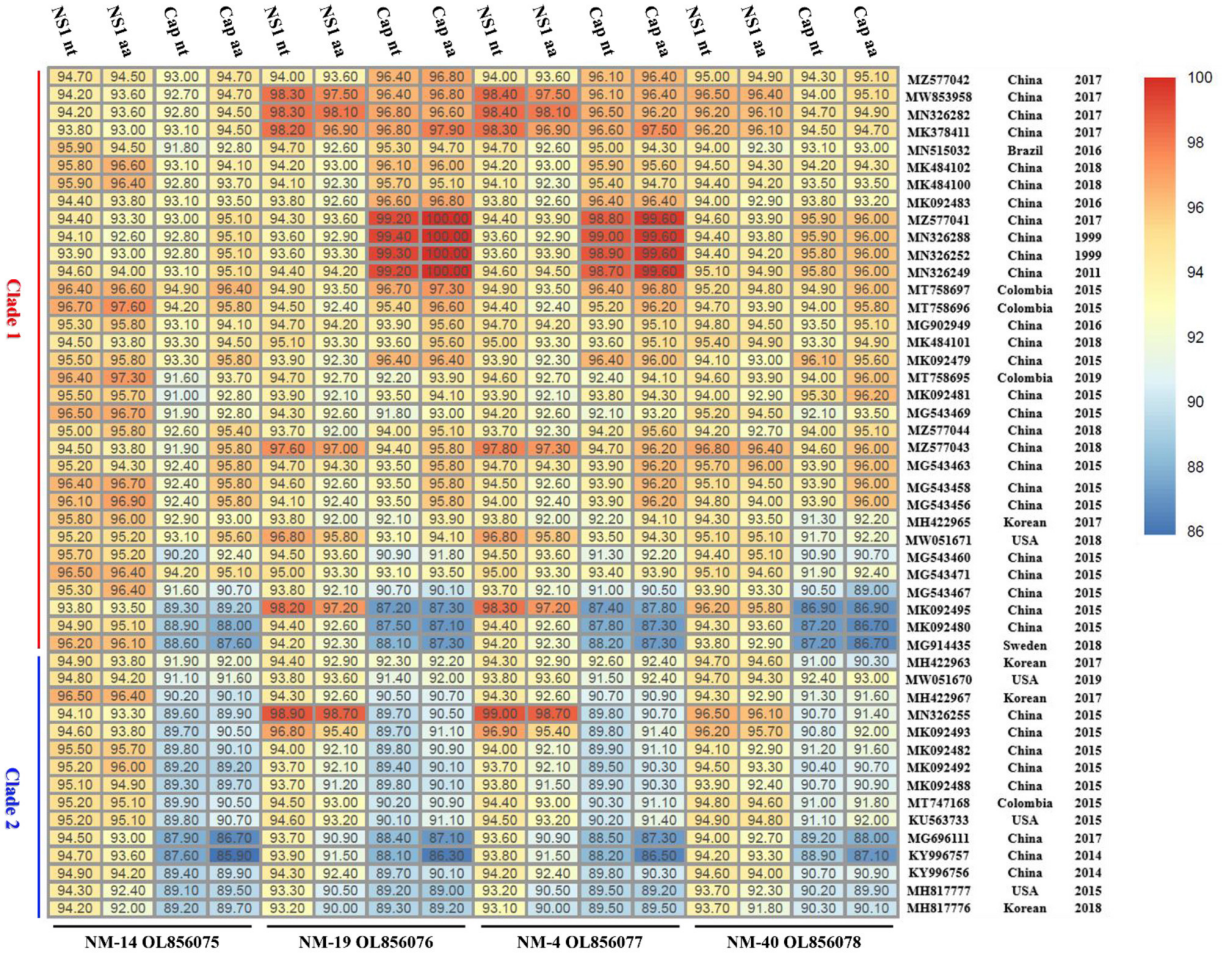
The nucleotide (nt) and amino acids (aa) similarity between the NS1/Cap of the four PPV7 isolates and that of the reference strains. The GenBank accession number, country, and year of the reference sequences are noted on the right of the figure. The sequences of the four strains shared 93.1–99.0% similarity in the NS1 nt, 90.0–98.7% similarity in the NS1 aa, 86.9–99.4% similarity in the capsid nt and 85.9–100% similarity in the capsid aa sequence compared with the 48 reference strains.

The nucleotide similarity between the genome of the four PPV7 strains identified in this study was 94.9–99.8%, with 93.1–99.7% similarity in the NS1 gene and 94.5–99.6% similarity in the capsid gene. In addition, the sequences of the four strains shared 80.0–96.9% similarity in the nearly complete genome, 93.1–99.0 similarity in the NS1 gene, and 86.9–99.4% similarity in the capsid gene sequence compared with the 48 reference strains (Figure 4). Specifically, alignments by the blast tool in NCBI showed that the capsid gene sequence of NM19 and PPV7-XZ7-1999 (MN326288) had the highest similarity (99.4%). The NS1 gene sequence of NM19 had the highest similarity with that of PPV7-HeN10-2015 (MN326255), up to 98.9%. However, the similarity between the NM19 NS1 gene sequence and that of PPV7-XZ7-1999 was 93.6%. The similarity between the capsid gene sequence of NM19 and that of PPV7-HeN10-2015 was only 89.7%. The capsid amino acid sequence of NM19 was completely identical to that of PPV7-JSYZ20170103-20 (MZ577041), GX22-1999 (MN632288), PPV7-XZ7-1999 (MN326252), and GD26-2011 (MN326249).

**Figure 5:**
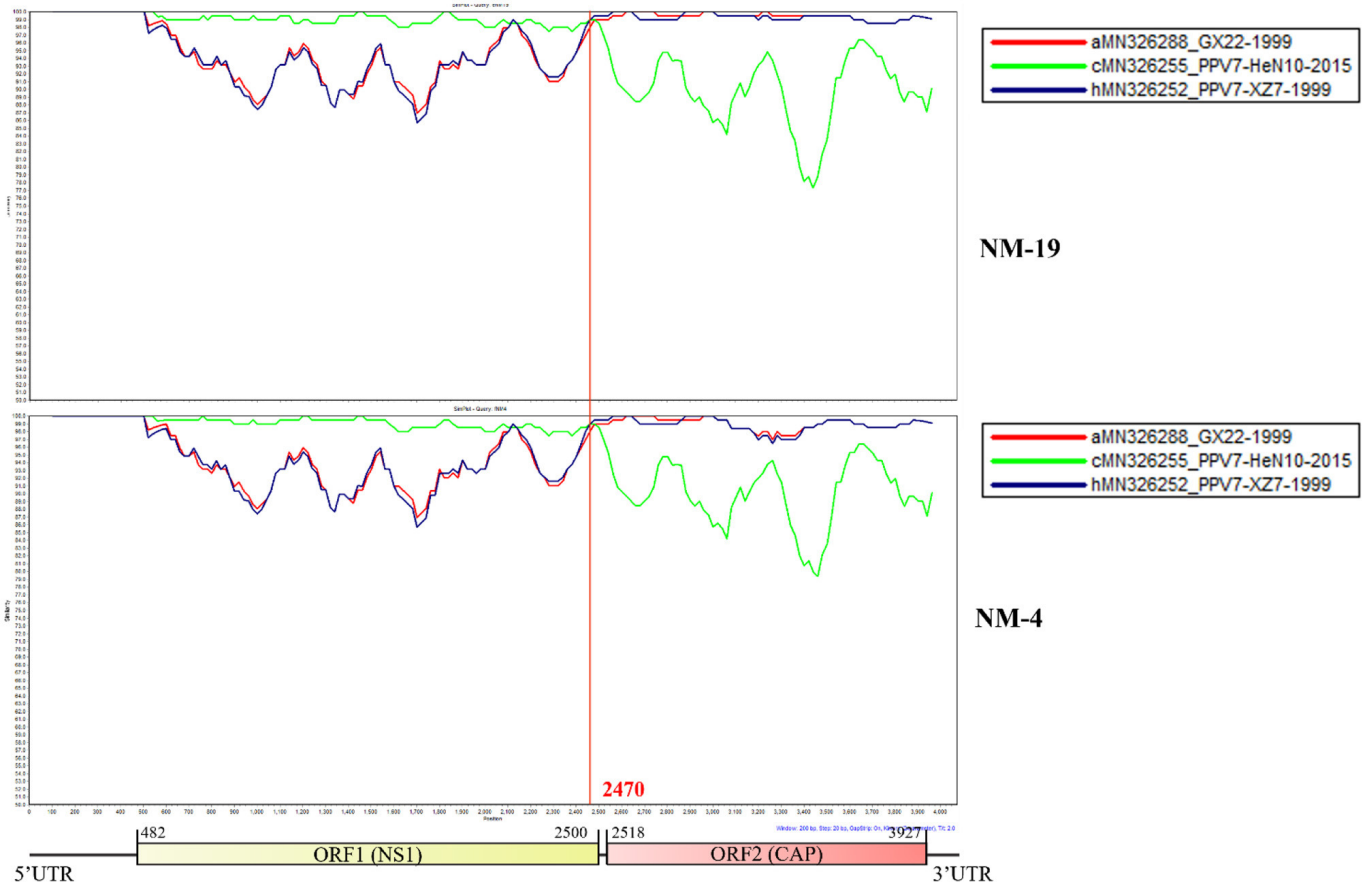
Similarity plot of whole genomes of different porcine parvovirus 7. The isolate NM-19 strain or NM-4 strain was set as the query strain, respectively. The vertical and horizontal axes represent the nucleotide similarity percent and nucleotide position (bp) of the alignment in the graph, respectively.

Recombination analysis showed that PPV7 NM-19 and NM-4 strains may be natural recombinant viruses with PPV7-HeN10-2015 (MN326255) like isolate as the major parental virus and PPV7-XZ7-1999 (MN326252) like isolate as the minor parental virus (Figure 5). The PPV7-HeN10-2015 (MN326255) like isolate provided the first half of about 2,470 bp in length, while the PPV7-XZ7-1999 (MN326252) like isolate provided almost the complete Cap gene with about 1,526 bp in length.

### Capsid Amino Acid Sequences Alignment of PPV7 Strains

**Figure 6:**
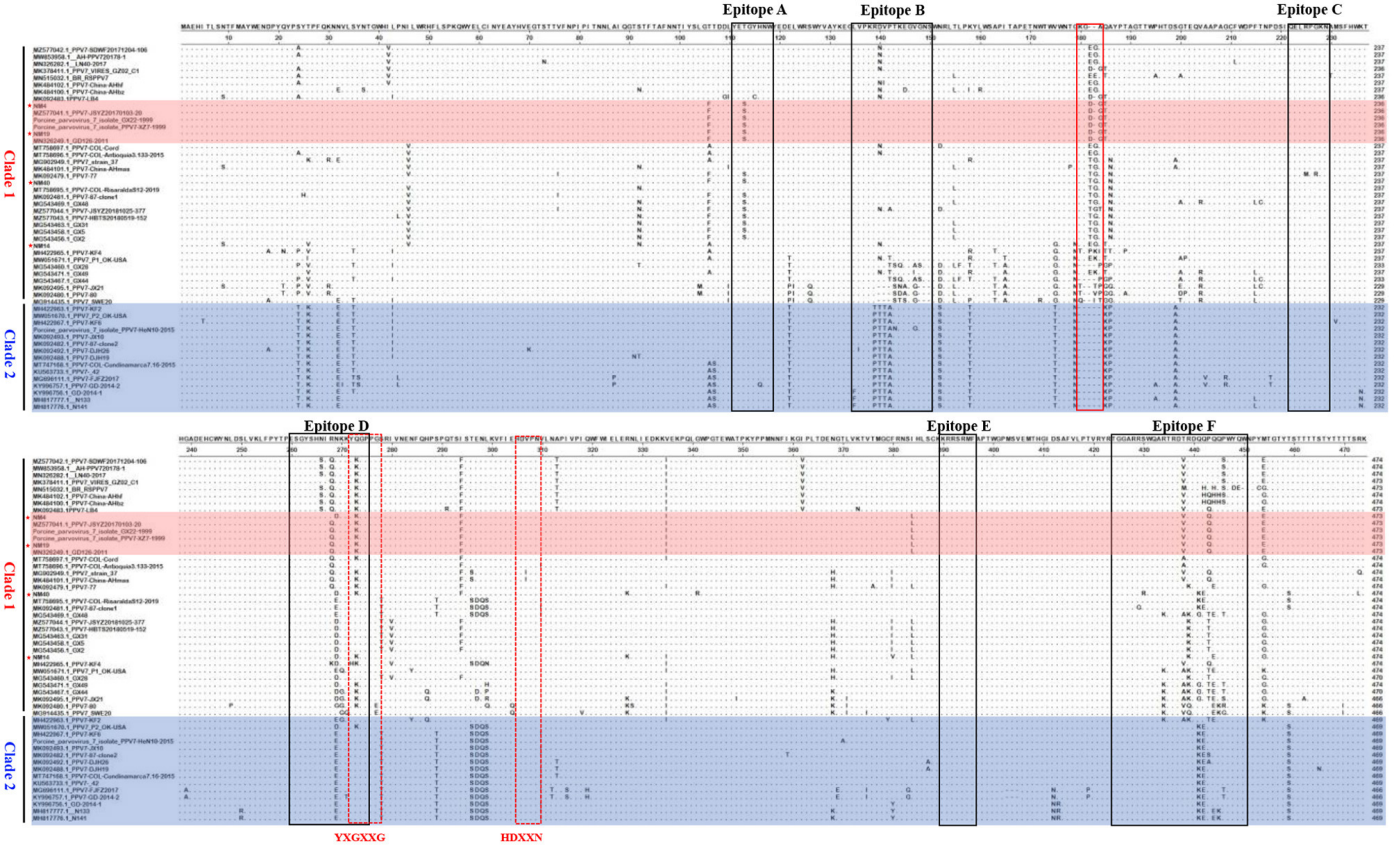
Alignment of Capsid amino acid sequences of PPV7 strains. The 6 linear B cell epitope regions (Epitope A~E) of PPV7 Capsid were marked with black box. The five-amino-acid deletion of PPV7 strains clustered in clade 2 was marked with a red solid box. The conserved amino acids of the Ca2+ binding loop (YXGXXG) and the catalytic residue (HDXXY) are marked with a red dotted box.

The capsid is the major structural protein of PPV7, as well as the major target of the host immune response. Through multiple sequence alignment of the capsid, we discovered that the capsid of all the PPV7 strains clustered in clade 2 had a five amino acids deletion at position 181–185aa (Figure 6). Further antigenicity testing by VaxiJen v2.0 showed that the 5-aa deletion did not affect the antigenicity of Cap because the antigenic score (0.4493) of NM4 Cap from clade 1 was the same as that of KF6 from clade 2. Moreover, a single amino acid deletion was found in the PPV7 strains NM-4 and NM-19 at position 183-aa. The same amino acid deletion also occurred in PPV7_VIRES_GZ02_C1(MK378411), PPV7-LB4 (MK092483), and PPV7- JSYZ20170103-20 (MZ25770411), which were all identified in China. In contrast, no amino acid deletion was found in NM-40 and NM-14.

**Figure 7:**
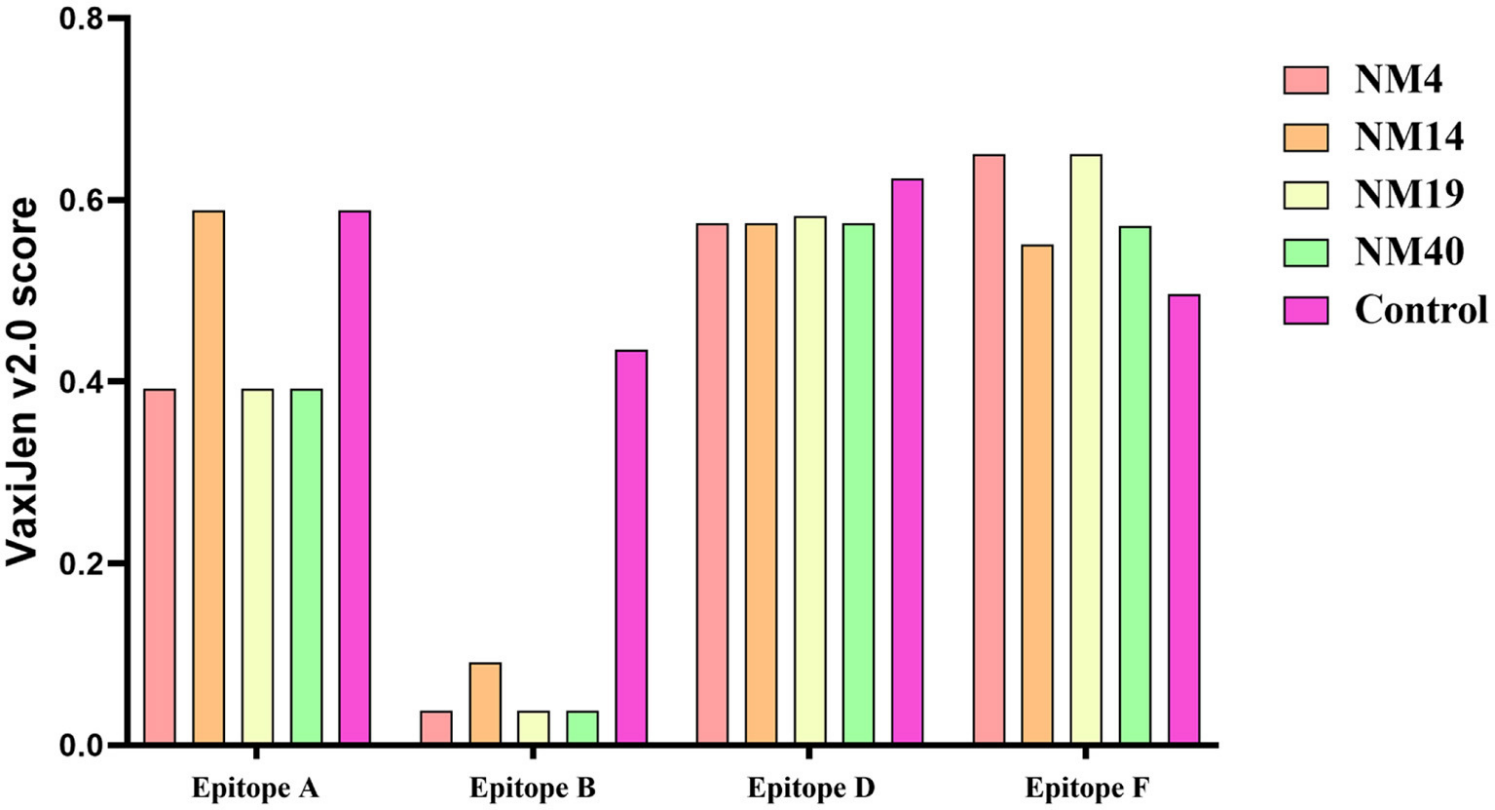
Antigenicity testing of epitopes A, B, D, and E in PPV7 strains was performed with the VaxiJen v2.0 server online tool. The control were the VaxiJen v2.0 scores of epitopes reported by wang et al. (24).

The potential Ca2+ binding loop 267YXGXXG272 and a catalytic-like motif 300HDXXN304 conserved in PPV7 were also found in the four strains identified in this study (Figure 4), with 267YKGPPG272 for the Ca2+ binding loop. Moreover, six linear B cell epitope regions of PPV7 capsid described by wang (24) were reviewed. Among them, six amino acids mutations were found in NM40 (T113S, E269D, Q273K, S430R, Q441K, and Q442E) and NM19 (T113S, R268Q, E269N, Q273K, T438V, and P443Q), meanwhile, 5 amino acids mutations were found in NM14 (T113S, E269D, Q273K, T438V, P443Q) and NM4 (T113S, E269D, Q273K, R439K, Q444E). VaxiJen v2.0 server online tool was utilized to evaluate the effect of these mutations on the isolate's antigenicity. Results showed that the T113S mutation in epitope A of NM4, NM19, and NM40 slightly decreased the antigenic score from 0.5888 to 0.3919 (Figure 7). Furthermore, compared with strains in clade 2, a “PTTA” to “RDVP” mutation was found in epitope B of NM4, NM19, NM40 (“RNVP” mutation in epitope B of NM14), and many other strains in clade 1, which significantly decreased the antigenic score from 0.4355 to 0.0380 (0.0911 for NM14) (Figure 7). In contrast, mutations in epitope D and epitope F have little influence on antigenicity.

## Discussion

Since its discovery in the United States in 2016 (3), PPV7 has attracted the attention of many researchers around the world. The prevalence of PPV7 in China has been examined by different researchers, and the prevalence varies. It is 32.8% (21/64) in Guangdong (12), 27.3% (105/385) in Guangxi (16), 20% (24/120) to 74% (148/200) in Anhui (17, 19, 25), 23% (48/209) in Hunan (18) and 15.4% (67/435) for samples collected in Xinjiang, Hebei, Shandong, Henan, Jiangsu, Anhui, Fujian and Guangdong (20). However, information concerning the prevalence and genetic evolution of PPV7 is still limited. Here, our investigation unveiled a positive rate of 28.72% (27/94) for PPV7 in the samples collected by our lab, indicating that PPV7 has already circulated in eastern Inner Mongolia Autonomous Region, China.

Currently, high coinfection rates of PPV7 and PCV2, or PPV7 and PCV3 have been reported. The coinfection rate of PPV7 and PCV2 was 17.4% (65/385) in Guangxi (16), and 17.5% (21/120) in Anhui (17), while the coinfection rate of PPV7 with PCV3 was 9.1% (11/120) in Anhui (17), and 24.76% (26/105) in the Northeast of China (26). Remarkably, a recent report revealed that the prevalence of PPV7 in PCV2-positive farms (65.5%, 19/29) was significantly higher than that in PCV2-negative farms (5.7%, 2/35) in Guangdong province (12). Moreover, another study from Poland showed that significantly higher PCV2 DNA loads were found in PPV7 positive sera in comparison with the PPV7 negative group, suggesting that PPV7 infections may contribute to increased PCV2 viremia in individual pigs (27). Similarly, it has been reported that the PPV7 positive rate in PCV3 positive serum was significantly higher than that in PCV3 negative serum. Furthermore, the copy number of PCV3 in PPV7 positive samples was significantly higher than that in PPV7 negative serum samples, indicating that PPV7 may be a co-acting factor for PCV3 replication (18). High coinfection rates of PPV7 and PCV2, or PPV7 and PCV3 were also observed in our study. Interestingly, in the five pig farms that are detected positive for PPV7, The PPV7 infection rates were significantly higher in PCV2 positive samples than that in PCV2 negative samples. However, there is no significant difference between the infection rates of PPV7 in PCV3 positive samples and in PCV3 negative samples. These results support the hypothesis that PCV2 could be a co-factor for PPV7 infection. In contrast, PCV3 may have no significant influence on PPV7 infection. Considering the limited sample size involved in our study, more studies involving larger sample size were needed to reveal the effects of PCV2 and PCV3 on PPV7 infection, which is helpful for PPV7 prevention and control. Regardless of the pathogenicity of PPV7, co-infection of PPV7 and PCV2 or PPV7 and PCV3 may further aggravate the clinical symptoms caused by PCV2 or PCV3 infection alone (11). Moreover, the association between PPV7 and PRRSV has been also suggested (28). The co-infection of multiple pathogens as co-factors for PPV7 is a serious challenge to pig farms, which needs more attention.

It has been suggested that PPV7 strains may have a common ancestor in 2004 [95% highest posterior density (HPD): 1986–2014] (24). In addition, many PPV7 sequences obtained from samples collected in the 1990 s in China are available in GenBank, indicating that PPV7 has circulated in China for more than 20 years. However, whether this novel virus emerged before the 2,000 s in eastern Inner Mongolia needs further investigation. Though some nearly complete genomes of PPV7 have been determined in some previous studies, more genetic information is still needed to characterize this novel virus. To investigate the molecular characteristics of PPV7 in eastern Inner Mongolia, four nearly complete genomes of PPV7 have been determined.

The PPV7 strains have been reported to be divided into two different genotypes based on the complete genome sequences of PPV7 with MCC tree construction, while NJ and ML trees based on NS1 and Cap didn't display similar clusters (24). Another phylogenetic analysis with ML tree based on the capsid nucleotide sequences of PPV7 strains were made by Diana et al. (14). Results showed that 52 PPV7 strains were divided into two different genotypes based on the presence or absence of a 5-aa deletion in the capsid protein. Besides, 21 PPV7 strains were divided into 3 clades based on the Cap gene nucleotide sequences in a recent study by a maximum-likelihood tree analysis (28). In our study, a total of 52 PPV7 strains were well-differentiated into two different clades by NJ tree based on the 5-aa deletion in Cap protein. Of note, Further analysis revealed that the five-aa deletion does not influence the PPV7 Cap antigenicity. Moreover, the four PPV7 strains identified in this study all belong to clade 1 with a 183-aa deletion in NM-4 and NM-19 compared with NM-14 and NM-40, indicating a different origin of these four strains. Whether aa deletions of the capsid protein have influence on the replication or pathogenicity of PPV7 needs further study.

Recombination is thought to be important for the generation of genomic diversity and the transmission of PPV7 from wild boars to domestic pigs, because several chimeric strains have been identified. PPV7 KF4 strain was a recombinant from the major parental virus 17KWB09 strain identified in Korean wild boars and the minor parental virus N133 strain from Korean domestic pigs (29). In addition, PPV7 HBTZ20 180519-152 strain from Chinese domestic pig in 2018 was identified as a recombinant with a JX15-like virus as the major parental virus and a JX38-like strain as the minor parental virus, both of which were isolated from Chinese wild boars in 2015(20). Interestingly, Simplot analysis showed that our NM-19 and NM-4 strains identified in 2020 may be recombinants with PPV7-HeN10-2015 (identified in 2015) as the major parental virus and PPV7-XZ7-1999 (identified in samples collected in 1999) as the minor parental virus. The recombinant event of PPV7 strains from parental viruses 16 years apart is an interesting finding, which may help to explain genetic evolution characteristics of PPV7.

The Cap protein of PPV is considered to be the main antigenic determinant that induces neutralizing antibody production. Therefore, it is used as the main antigen for anti-PPV subunit vaccine development (15, 30). Since it has been reported that changes in the capsid amino acid sequence of PPV could affect receptor binding, pathogenicity, and antigenicity (31, 32). The study of the capsid amino acid sequence of the newly discovered PPV7 is of great significance for antigenicity analysis and vaccine development. Of note, the capsid amino acid sequence of NM19 was completely identical to that of several PPV7 strains collected in 1999, 2011, and 2017 in different regions of China indicating that PPV7 Cap maintains relatively stable with no significant temporal and geographic correlation.

In total, six potential linear B-cell epitopes in PPV7 capsid located in loops were predicted with Bepipred 2.0 by Wang et al. (24) in 2020. Due to the unsuccessfully 3D structure construction of PPV7 capsid, it is not clear whether these B cell epitopes are located on the capsid surface. However, as the B cell epitopes of PPV1 play an important role in viral infection and immunogenicity due to their location on the capsid surface, it is speculated that the amino acid mutations of PPV7 B cell epitopes may affect receptor binding and immunogenicity. Through multiple alignments of capsid amino acids, we found that the aa sequences divergence of the B cell epitopes in calde 1 strains was significantly higher than that in clade 2 strains. Furthermore, compared with clade 2 strains, a “PTTA” to “RDVP/RNVP” mutation that significantly decreased the antigenicity of epitope B occurred in clade 1 strains, supporting the speculation that PPV7 may use antigenic shift to escape host immune responses (24).

In conclusion, we detected the infection of PPV7 in eastern Inner Mongolia Autonomous Region, China for the first time. The four nearly full-length sequence was amplified, and the molecular characteristics of PPV7 were analyzed. Our results suggest that PPV7 has been prevalent in eastern Inner Mongolia with gene variants. More research is needed to elucidate the prevalence, genetic evolution, and pathogenicity of this novel virus.

## Data Availability Statement

The datasets presented in this study can be found in online repositories. The names of the repository/repositories and accession number(s) can be found in the article/supplementary material.

## Ethics Statement

The authors confirm the ethical policies of the journal, as mentioned in the journal's author guidelines. Animal materials used in the current study were received as routine diagnostic submissions under the permission of Inner Mongolia Minzu University. The authors also confirm that Ethical approval was not required for the use of animal materials received for diagnostic purposes.

## Author Contributions

SW, JY, ZC, and JZ conceived and designed the experiments. SW, XL, XM, and FD carried out the PPV7, PCV2, PCV3, and PCV4 detection and genome amplification of PPV7. SW and YS contributed to writing and revision of the manuscript. KL, JH, and XH prepared the samples. All authors read and approved the final manuscript.

## Funding

This work was supported by the following Grants: The Open Funding Project of Brucellosis Prevention and Treatment Engineering Research Center of Inner Mongolia Autonomous Region (Nos. MDK2019082 and MDK2021078). Doctoral Funding of the Inner Mongolia Minzu University (Nos. BS584 and BS583). Young Scientific and Technological Talents in Inner Mongolia (No. NJYT22053). Key Research and Development Program in Inner Mongolia Autonomous Region (Nos. 2021ZD001301 and 2019ZD006).

## Conflict of Interest

The authors declare that the research was conducted in the absence of any commercial or financial relationships that could be construed as a potential conflict of interest.

## Publisher's Note

All claims expressed in this article are solely those of the authors and do not necessarily represent those of their affiliated organizations, or those of the publisher, the editors and the reviewers. Any product that may be evaluated in this article, or claim that may be made by its manufacturer, is not guaranteed or endorsed by the publisher.
